# Intolerance of Uncertainty: A Temporary Experimental Induction Procedure

**DOI:** 10.1371/journal.pone.0155130

**Published:** 2016-06-02

**Authors:** Oriana Mosca, Marco Lauriola, R. Nicholas Carleton

**Affiliations:** 1 Department of Social and Developmental Psychology, University of Rome ‘Sapienza’, Rome, Italy; 2 The Anxiety and Illness Behaviour Laboratory, University of Regina, Regina, Saskatchewan, Canada; Universidad de Alicante, ITALY

## Abstract

**Background and Objectives:**

Intolerance of uncertainty (IU) is a trans-diagnostic construct involved in anxiety and related disorders. Research focused on cross-sectional reporting, manipulating attitudes toward objective and impersonal events or on treatments designed to reduce IU in clinical populations. The current paper presents an experimental procedure for laboratory manipulations of IU and tests mediation hypotheses following the Intolerance of Uncertainty Model.

**Methods:**

On pre-test, undergraduate volunteers (Study 1, *n* = 43;68% women. Study 2, *n* = 169;83.8% women) were asked to provide an idiosyncratic future negative life event. State-IU, Worry, Positive and Negative Affect were assessed after that a standardized procedure was used to identify event’s potential negative consequences. The same variables were assessed on post-test, after that participants were asked to read-through increasing and decreasing IU statements.

**Results:**

Temporary changes on IU were consistently reproduced in both studies. Participants receiving increasing IU instructions reported greater state-IU, Worry and Negative Affect than those receiving decreasing IU instructions. However, this latter condition was not different from a control one (Study 2). Both studies revealed significant indirect effects of IU induction instructions on Worry and Negative Affect through state-IU.

**Limitations:**

Both studies used undergraduate psychology students samples, younger than average population and predominantly female. Experimental manipulation and outcome measures belongs to the same semantic domain, uncertainty, potentially limiting generalizability.

**Conclusions:**

Results supported the feasibility and efficacy of the proposed IU manipulation for non-clinical sample. Findings parallel clinical research showing that state-IU preceded Worry and Negative Affect states.

## Introduction

Intolerance of Uncertainty (IU) was first delineated as a construct of interest for “representing cognitive, emotional, and behavioral reactions to uncertainty in everyday life situations” [1]. The construct was focused initially on understanding worry and generalized anxiety disorder [2–4]; however, recent evidence now suggests IU represents a critical trans-diagnostic construct for understanding neuroticism [5, 6–7, 3, 8, 9]. There is also broad evidence [10] that fearful responses to unknowns, typically measured as IU, are important for models of emotion [11], attachment [12], and personality [13]. Accordingly, the definition has been revised several times [5], with the most recent stating that IU is “an individual’s dispositional incapacity to endure the aversive response triggered by the perceived absence of salient, key, or sufficient information, and sustained by the associated perception of uncertainty” [7].

The broad importance of fearing the unknown and IU led to clinical research studies exploring interventions targeting the IU construct [14, 15, 5, 7, 8]. Most research has focused on reducing worry in patients with GAD, following the Intolerance of Uncertainty Model [16]. In the IUM, uncertainty drives worry, perceived as an effective strategy to cope with uncertainty, but producing a worrying chain. Effective treatments for reducing IU have included individual [17,18] and small group therapy sessions [19] using a cognitive-behavioral approach specifically targeting IU that facilitates re-evaluation of positive beliefs about worry, cognitive exposure, and problem-solving. The treatments reduced IU, causally related to changes in worry [19,17,18], and reduced somatic symptoms, general anxiety and depression [18,19], for up to 2-years [19].

Besides clinical research, there is mounting evidence that IU is an individual difference variable that biases one’s appraisal of outcomes and probability in behavioral decision making. For instance, Jacoby et al. [20] have shown that trait IU was associated with Negative Affective reactions to uncertainty using a probabilistic inference task, in which people have to decide from which of two jars a series of beads has been drawn. Furthermore, Luhmann, Ishida, & Hajcak [21] showed that higher trait IU was associated with greater preferences for risky options that might provide an immediate smaller reward than for risky options that might confer a delayed greater reward. Thus, trait IU predicted a disadvantageous risky choice pattern, not merely risk perception or distress with uncertainty, in keeping with behavioral studies on trait anxiety and performance in the Iowa Gambling Task [22–24]. More recently, Carleton et al. [10] found a significant positive association of trait IU with performance in a Risky-Gains Task, in which research participants had very limited time to decide between a sooner non-risky option and two risky options presented later. Specifically, high trait IU individuals preferred an immediate safe reward rather than waiting for risky options that could be more advantageous in terms of expected returns. As a whole, this literature has shown that individual differences in IU affected probability judgment and risky choices.

### Intolerance of uncertainty manipulation in laboratory settings: state of the art

Inducing temporary changes in IU state is a challenging issue, especially for non-clinical participants in experimental settings. Treatment studies to date typically involved clinical samples characterized by high levels of IU. In addition, time-, skill-, and effort-demanding cognitive behavioral therapy techniques were used in these studies, which would be difficult tools for studying IU in non-clinical designs (e.g., social or cognitive psychology experiments). Instead, researchers studying IU would benefit from being able to experimentally manipulate the construct and associated behaviors irrespective of clinical status. Initial laboratory-based manipulations have been devised to study causal links between IU and higher order cognitive processing [18,25].

Ladouceur and colleagues [18] built an experimental manipulation of IU using a computerized roulette game, in which the uncertainty about the outcome of the game was manipulated. Participants were randomly assigned to one of two experimental conditions: increased or decreased uncertainty [18]. Each participant was given $20 to play with the computerized game and the experimenter informed participants that their winnings would be donated to a fictitious Foundation if each participant’s winnings were equal to or greater than the initial amount given to the participants. If the final amount was less than the initial $20, nothing would be donated to the Foundation. Participants in the “increased uncertainty” group received information throughout the game that their chances of winning (probability) were low (i.e., the experimenter said: “your chances of winning are much lower than those used in previous studies with this task”). In contrast, the “decreased uncertainty” group received information that their chances were high. Participants in the high uncertainty condition reported significantly more worry; however, the authors did not assess baseline IU or worry before the experimental manipulation, which was an important experimental limitation. In addition, a computerized roulette game about a fictitious Foundation may not be sufficiently ecologically valid relative to clinical research using personally relevant events [17, 19, 18]. Finally, the manipulations used focused on probabilities and therefore might be manipulations of perceived risk rather than uncertainty.

Grenier and Ladouceur [25] bridged the gap between non-clinical and clinical experimental designs by manipulating IU with a two-stage crossover experimental design. On pre-test, participants were invited to reveal a negative life event that might occur in the future (e.g., be refused to higher education). A “Vertical Arrow Technique” [26] was then used to help participants identify several negative sequelae. Then, participants were asked to imagine that they ingested a drug that had provoked an unpredictable effect (other than relieve an headache or to alleviate a bad cough) and then assigned to one of two alternative IU manipulation conditions. One week later, participants repeated the same procedure, but they were assigned to the other IU manipulation condition. The results supported the manipulations as effective at increasing IU, but only for participants who reported relatively low state anxiety at pre-test. In contrast, the manipulations were effective at decreasing IU for all participants, which was in line with earlier clinical findings [17, 19, 18].

Grenier and Ladouceur [25] might be extended in two important ways. First, we suggest that focusing on a personal negative future life event, rather than an event posited by the research team (i.e., imagining that they had ingested a drug), and then manipulating perceptions of that idiosyncratic event, could produce a more salient, coherent, and effective manipulation. Second, Grenier and Ladouceur [25] used a counterbalanced crossover requiring repeated checks of manipulation, mood, and IU, as well as trained clinical psychologists for running the Vertical Arrow Procedure; in contrast, the proposed procedure uses a standardized and abridged variation of Grenier and Ladouceur [25] to facilitate use in several non-clinical settings (e.g., social or cognitive psychology experiments).

## Study 1

The first study was designed to pilot test the feasibility, and potential efficacy, of different IU inducing instructions on short-term worries and IU levels in laboratory setting. The experimental manipulation replicated Grenier and Ladouceur [25], but attempted to extend their procedure by 1) having participants focus on an idiosyncratically-selected potential negative event–instead of a neutral event; and 2) having participants self-administer the manipulation.

### Methods

#### Participants

Participants included 43 undergraduate volunteers (68% women), recruited in the University library to attend a two-session study. Participants were prescreened to determine whether any report exceeding the psychological distress cut-off score on the Hospital Anxiety and Depression Scale [27] and the presence of a potential clinical sub-sample. No participant exceeded the cut-off scores of the scale. Session 1 occurred during the morning and session 2 during the afternoon of the same day. Session 1 involved the pre-test, whereas session 2 involved the experimental manipulation and the post-test. Participants were compensated with a 5€ phone credit card awarded after they completed the second session. The study was approved by the ethical committee of the Department of Social and Developmental Psychology, University of Rome “Sapienza” (#62-CED-01).

### Measures

#### Intolerance of Uncertainty Scale, Short Form

The IUS-12 [28] was back-translated into Italian by the authors for use in the current study. The IUS-12 is a short version of the original 27-item Intolerance of Uncertainty Scale [1] and measures responses to uncertainty. The 12 items are rated on a 5-point Likert-type scale ranging from 1 (*not at all characteristic of me*) to 5 (*entirely characteristic of me*). The IUS-12 total score can represent trait IU [29], which is how it was used in the current study. In the current study the internal consistency for the total score was .97.

#### Worry and Intolerance of Uncertainty Questionnaire

The Worry and Intolerance of Uncertainty Questionnaire (Q-III) [25] is a scale specifically developed to assess fluctuations in the level of IU and Worry. In Grenier and Ladouceur [25], the QIII was comprised of 9 items, each of which required participants to write a number from 0 (i.e. = no) to 12 (i.e. = extremely) in empty blanks within each item to express their response. In this study, we used a more standard Likert-type scale aside of each item 0 (*I totally disagree with the item*) to 12 (*I totally agree with the item*). In addition, two control items (i.e., item 1, “When I think that the negative events mentioned above may indeed occur I consider this possibility acceptable” and item 4, “The experimenter is able to assure me (100%) that the negative events mentioned above will never occurr”) [25] were removed due to zero variance on a pilot administration. The Q-III administered in this study was indeed comprised of 7 items only, of which four assessed state-IU (i.e.: “I agree with the following statement: I always want to know what my future sets aside for me”) and three assessed Worry (i.e. “I think that the negative events mentioned above worry me”). The internal consistency for the state-IU and Worry at pre-test were .83 and .86, respectively. At post-test the coefficients were .83 and .92, respectively.

#### Positive and Negative Affect Schedule

The PANAS [30, 31] is a 20-item measure that assesses positive and negative affect states on two 10-item subscales related to positive (e.g., cheerful) and negative emotions (e.g., sluggish), respectively. For each item, participants were asked to describe how they felt “at the moment” on a 5-point Likert-type scale, ranging from 1 (very slightly or not at all) to 5 (very much). Positive and Negative Affect scores were obtained summing up the respective items. The internal consistency for negative and Positive Affect at pre-test was .91 and .85, respectively. At post-test the coefficients were .93 and .87, respectively.

### Procedures

#### Session 1. Pre-test

Participant condition assignment was based on alternating recruitment order (e.g., the first participant was assigned to the increasing IU condition, the second to the decreasing condition, continuing alternating throughout collection). Participants assigned to the increasing IU condition were expected to report higher scores on measures of worry and IU than those assigned to the decreasing IU condition. At arrival for session 1 of the study each participant was acquainted with the expected duration, general procedure, and compensation for participation. The study was presented as related to individual differences in personality and cognition, with no specifics provided about the intended IU manipulation. Participants were informed that they could leave at anytime without losing their compensation. All participants who arrived at the lab provided informed consent to participate and all participated in both sessions. All questionnaires were completed as paper and pencil tools.

During session 1, participants completed the pre-test questionnaires. Like Grenier and Ladouceur [25], participants were asked to provide an idiosyncratic negative life event that might occur in the future (e.g., being fired from work; failing an exam). A standardized self-administered VAT procedure was used to identify several potential negative consequences that would result from the provided negative event. The self-administration involved a hierarchical schema presented to each participant on an A3 size paper (see S1 Appendix). On the first level, a box allowed the participant to write down the potential negative future outcome. The first box was connected via downward arrows to three smaller boxes on a second level, each of which could be filled in with a potential consequence, if the first level event occurred. Any participants unable to provide three consequences from the top level event were instructed to leave one or more boxes empty. Each second level box was subsequently connected via downward arrows to three boxes at a third level, each of which could be filled with a potential consequence, if any second level event occurred. Again, participants could leave one or more boxes empty. After the diagram was completed the modified Q-III and the PANAS were administered to assess state-IU and mood.

#### Session 2. Experimental manipulation

Session 2 occurred during the afternoon of the same-day as Session 1. Each participant was asked to recall the potential future negative event that he/she provided during session 1; thereafter, participants were assigned to an increasing (*n* = 22) or decreasing (*n* = 21) IU condition. Participants assigned to the increasing IU condition were asked to read and repeat aloud the all the statements reported by Grenier and Ladouceur [25], such as, “Concerning the negative event it's difficult not know what will happen”. Participants assigned to the decreasing IU condition were asked to read and repeat aloud statements such as, “It doesn't bother me to not know what will happen to me” (detailed in S2 Appendix). Statements were presented individually and sequentially on a desktop computer with each participant’s idiosyncratic negative life event affixed to the top of the screen and used as memory cue.

#### Post-test

The QIII and PANAS were administered after the experimental manipulation, therein assessing participant reactions to each of the two IU conditions. The post-test administrations allowed for comparisons with the pre-test scores on the QIII and PANAS, therein determining the effectiveness of the manipulation. Lastly, participants were debriefed regarding the experimental hypotheses.

### Statistical Analyses

The data were preliminarily checked for univariate outliers. Descriptive statistics for study variables used for outliers detection and testing assumptions are reported in Table 1 (Panel a). Three cases were identified and removed from subsequent analyses. Parametric assumptions for ANCOVA were tested, including normal distribution and homoscedasticity. Minor violations were detected and the analyses re-run based on non-parametric analyses [32,33]. Mediation analyses were carried out by INDIRECT SPSS procedure. The statistical significance of indirect effects was based on bootstrap bias corrected confidence intervals [34]. More details about statistical procedures are provided in S3 Appendix.

**Table 1:** Descriptive statistics and tests of assumptions for Study 1 (Panel a) and Study 2 (Panel b) variables.

**Panel a)**	**Pre-test**	**M**	**SD**	**Sk**	**K**	**S-W**	**p-level**	**L**	**p-level**
	Positive Affect	3,01	0,57	-0,03	-1,00	0,96	Ns	0,24	ns
	Negative Affect	1,67	0,68	1,14	0,79	0,87	p < .01	0,18	ns
	IU	4,84	2,09	0,47	-0,68	0,95	p = .09	0,57	ns
	Worry	7,10	2,15	-0,17	-0,35	0,98	Ns	0,61	ns
	Posttest								
	Positive Affect	3,02	0,58	0,13	-0,46	0,98	Ns	0,15	ns
	Negative Affect	1,67	0,68	0,91	-0,21	0,86	p < .01	1,88	ns
	IU	3,85	1,65	0,63	-0,36	0,94	p < .05	1,55	ns
	Worry	5,30	2,91	0,15	-1,06	0,95	p = .09	0,41	ns
**Panel b)**	**Pre-test**	**M**	**SD**	**Sk**	**K**	**S-W**	**p-level**	**L**	**p-level**
	Positive Affect	58,47	19,53	-0,33	-0,27	0,98	p < .05	2,14	ns
	Negative Affect	35,55	20,87	0,29	-0,68	0,97	p < .01	0,39	ns
	IU	5,64	2,01	0,18	-0,33	0,99	Ns	0,21	ns
	Worry	7,60	2,46	-0,39	-0,53	0,97	p < .01	0,51	ns
	Posttest								
	Positive Affect	57,98	19,03	-0,25	-0,14	0,98	Ns	1,18	ns
	Negative Affect	33,40	20,90	0,42	-0,53	0,97	p < .01	1,82	ns
	IU	5,52	2,10	0,11	-0,34	0,99	Ns	1,20	ns
	Worry	6,87	2,45	-0,24	-0,40	0,99	Ns	1,26	ns

Note: Sk = Univariate Skewness; K Univariate Kurtosis; S-W = Shapiro-Wilks normality test; L = Levene's test of variance homogeneity.

## Results

Participants in increasing IU or decreasing IU conditions were compared on sex ratios using chi-square tests and on IUS-12 scores using independent-samples *t*-tests. No significant differences were found between the two conditions. Likewise, the two conditions did not differ on pre-test scores for all variables but only for Positive Affect, *t*(38) = 2.08, *p* > .05, *η*^2^ = .10 (average scores 3.18 vs. 2.81 for decreasing and increasing IU, respectively). Post-test scores were largely correlated with pre-test ones (*r*s .85, .73, .89 and .73 for state-IU, Worry, Positive and Negative Affect, respectively). Differences and autocorrelations were accounted for in the data analyses [35].

**Fig 1:**
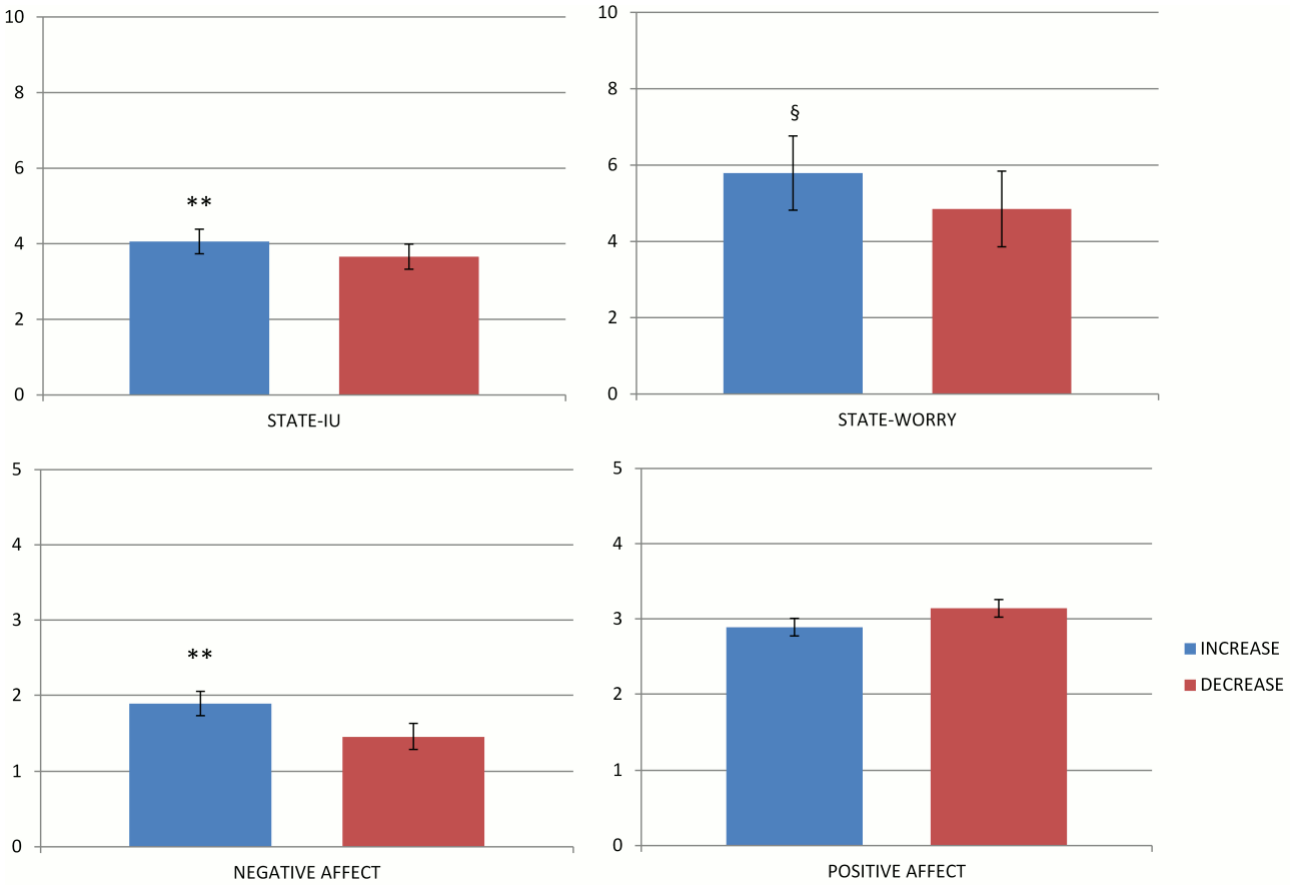
ANCOVA by condition.

As expected, ANCOVAs by condition resulted significant effects of covariates (i.e., pre-test scores) on dependent variables (i.e., post-test scores) (all *p*s < .001). The condition effect controlling for covariates was fully significant for state-IU, *F*(1,37) = 13.93, *p <* .001, partial η^2^ = .27, and Negative Affect, *F*(1,37) = 17.28, *p <* .001, partial η^2^ = .32; marginally significant for Worry, *F*(1,37) = 2.23, *p* = .14, partial η^2^ = .06; and no significant at all for Positive Affect (partial η^2^ = .03). Participants in the increased IU condition reported higher state-IU, Worry, and Negative Affect than those in the decreased IU condition (Fig 1). Because we detected marginal violations of normality assumptions (see Table 1, Panel a), we re-examined our hypothesis by non-parametric analyses to check whether and to what extent the results obtained from previous analyses were biased. Since Parametric ANCOVA and nonparametric RANCOVA gave nearly identical results (*F* = 13.78, *p <* .001 for state-IU; *F* = 17.56, *p <* .001 Negative Affect; *F* = 2.28, *p* = .14 for Worry) we concluded that “the assumptions underlying the usual analysis of variance are likely to be reasonable and the regular parametric analysis valid” [36]. These findings supported the main hypothesis that IU induction affected state-IU, Worry and Negative Affect for participants.

**Fig 2:**
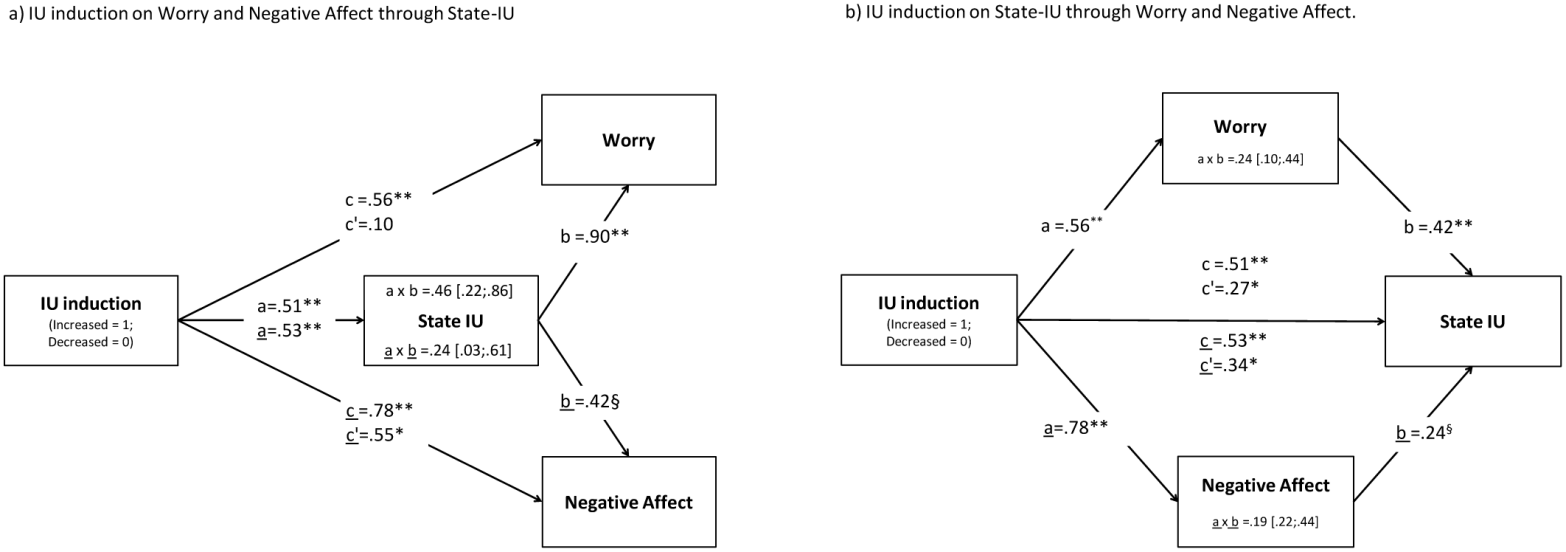
Mediation analysis.

Consistent with the IUM [2,37], IU induction appeared to trigger a worrying chain through increased state-IU. Accordingly, we tested indirect effects of IU induction on Worry and Negative Affect post-test scores through state-IU, controlling for pre-test scores (Fig 2a). The analysis was overall statistically significant for both Worry, *R*^2^ = .77, *F*(4,35) = 29.27, *p* < .001, and Negative Affect, *R*^2^ = .72, *F*(4,35) = 22.18, *p* < .001. Importantly, state-IU fully mediated the relation between IU induction and Worry (*c* = .56; *p* < .01 and *c’* = .10; *p* = ns) and partially mediated that between IU induction and Negative Affect (*c* = .78; *p* < .01 and *c’* = .55; *p* < .05). Since the putative mediating variable (i.e., state-IU) was measured at the same time as post-manipulation Negative Affect and Worry, an alternative account of experimental findings could be that IU induction affected state-IU through Worry or Negative Affect. As such, alternative mediation analyses were carried out in which mediator and dependent variables were reversed (Fig 2b). Again the analysis was overall statistically significant for both Worry, *R*^2^ = .89, *F*(4,35) = 73.02, *p* < .001, and Negative Affect, *R*^2^ = .83, *F*(4,35) = 45.70, *p* < .001. The indirect effects for IU induction on state-IU through Worry and Negative Affect were also significant (all *p-s* > .05). However, mediation relations were only partial (*c* = .51; *p* < .01 and *c’* = .27; *p* < .05 for Worry as mediator; *c* = .53; *p* < .01 and *c’* = .34; *p* < .05). Based on these findings we could not rule out the alternative account that IU induction affected state IU through Worry or Negative Affect. This issue is addressed in the following study.

## Study 2

Study 1 supported IU induction as significantly influencing state-IU and therein Worry and Negative Affect; however, there were three major limitations. First, there was no control group. Second, results of the mediation analyses were comparable for alternative models (i.e., IU-induction effects on state Negative Affect through state-IU vs. IU-induction effects on state-IU through Negative Affect). Last, the time’s interval which separated the induction of IU occurred in the pre-test (morning) and the manipulation of IU occurred at the post-test (afternoon) was relatively small, increasing the possibility of carryover effects. Study 2 was designed to replicate Study 1 as well as to resolve these limitations by including a control condition, setting a longer time interval between sessions and comparing two mediation models using confirmatory path analyses.

### Methods

#### Participants

Participants were 169 undergraduate psychology students (82.8% women, ages ranging from 19 to 51, *M* = 24.83; *SD* = 4.74) who participated in the two-session study. Unlike Study 1, Session 2 was carried out 7-to-14 days after Session 1 (*M* = 9.98 days; *SD* = 2.15 days), in which a pre-test was done, including assessment of trait IU and filling in the VAT sheet. Students participated in exchange for course credits. The study was approved by the ethical committee of the Department of Social and Developmental Psychology, University of Rome “Sapienza” (#62-CED-01).

#### Procedure and materials

All questionnaires were completed as paper and pencil tools. Pre-test generally followed the procedures outlined for in Study 1 except for: 1) only the IUS-12 was administered before the VAT; 2) four mood adjectives were administered along the Q-III after the VAT on pre-test and after taking IU induction instructions on post-test. Switching from PANAS to mood adjectives made the experimental procedure less cumbersome for both experimenter and participants. Moreover, the four adjectives (i.e., Happy, Sad, Calm, Anxious) were selected to represent high-low arousal unpleasant-pleasant emotions according to the Russell’s circumplex model of affect [38]. Participants rated how each adjective described their feelings “at the moment” on a Visual Analog Scale from 0 (Not at all) to 100 (Completely). Positive and Negative affective states were operationalized by averaging Happy and Calm ratings as well as Sad and Anxious ratings, respectively. This procedure yielded Positive and Negative Affect scores that are balanced in terms of affect activation [39]. Additional procedural variations included: 1) a longer time-interval between Session 1 and Session 2 to reduce carryover effects that could potentially confound the IU induction effects with the IU manipulation effects; 2) full review of the VAT before taking IU induction instructions to refresh participant memory for the negative event and uncertain consequences; and 3) reading statements off of the computer screen instead of repeating them aloud to better focus on the manipulation statements. The QIII and Mood Adjectives were administered post-manipulation to assess participant reactions to the different IU conditions. Participants were randomly assigned using the MS Excel random generator function to an increasing IU condition (*n* = 59), to a decreasing IU condition (*n* = 58) or to a control condition (*n* = 52). In the control condition participants were asked to read descriptive statements unrelated to the negative event (e.g., “Everest is earth's highest mountain. Its peak is 8,848 meters above sea level”; details in S2 Appendix).

#### Statistical analyses

As in Study 1, the data were preliminarily checked for univariate outliers. Two cases were identified and removed from subsequent analyses. Parametric assumptions for ANCOVA were substantially met (see Table 1, panel b). For the sake of prudence, and consistent with Study 1, research hypotheses were re-examined based on non-parametric tests. Mediation relations were tested through confirmatory path analysis models with manifest variables carried out by EQS 6.1 [40]. More details about statistical procedures are provided in S3 Appendix.

## Results

**Fig 3:**
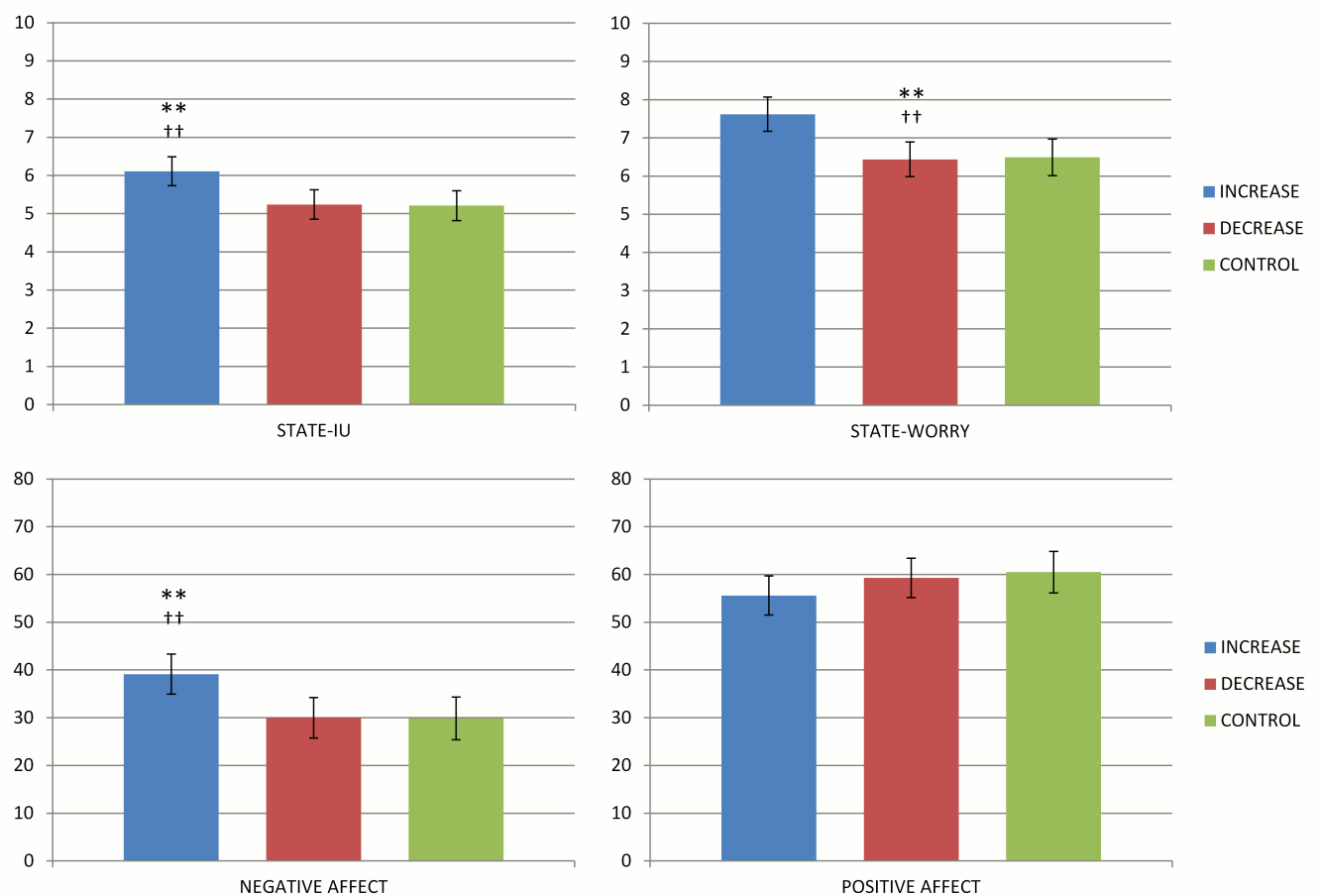
ANCOVA by condition.

A preliminary descriptive analysis of the IUS-12 scores collected at pre-test revealed that there were no significant differences between participants later assigned either to the increased, decreased IU or control conditions. Likewise, the three conditions did not differ on pre-test scores for all variables. As in Study 1, post-test scores were largely correlated with pre-test ones (*r*s .67, .66, .52, and .58 for state-IU, Worry, Positive Affect, and Negative Affect, respectively) and their effects on post-test scores were significant in ANCOVAs by condition (all *p*s < .001) except for Positive Affect. The time interval occurring between Session 1 and Session 2 had no significant effect on any dependent variable. Importantly, the condition effect was fully significant for all variables except for Positive Affect (i.e., state-IU, *F*(3,163) = 8.05, *p <* .001, partial η^2^ = .13; Negative Affect, *F*(3,163) = 6.46, *p <* .001, partial η^2^ = .11; Worry, *F*(3,163) = 5.48, *p <* .001, partial η^2^ = .09). Participant in the increased IU condition experienced higher IU, Worry, Negative Affect and Positive Affect than those in the decreased IU condition (Fig 3); however, participants in decreasing IU condition resulted in post-test levels of state-IU, Worry and Negative Affect scores that were as large as controls. Like Study 1, parametric ANCOVA and nonparametric RANCOVA gave nearly identical results (*F* = 7.10, *p <* .001 for state-IU; *F* = 6.29, *p <* .001 Negative Affect; *F* = 5.69, *p* < .001 for Worry). Accordingly, instructions to increase IU were more effective determinants of change than instructions to decrease IU.

**Fig 4:**
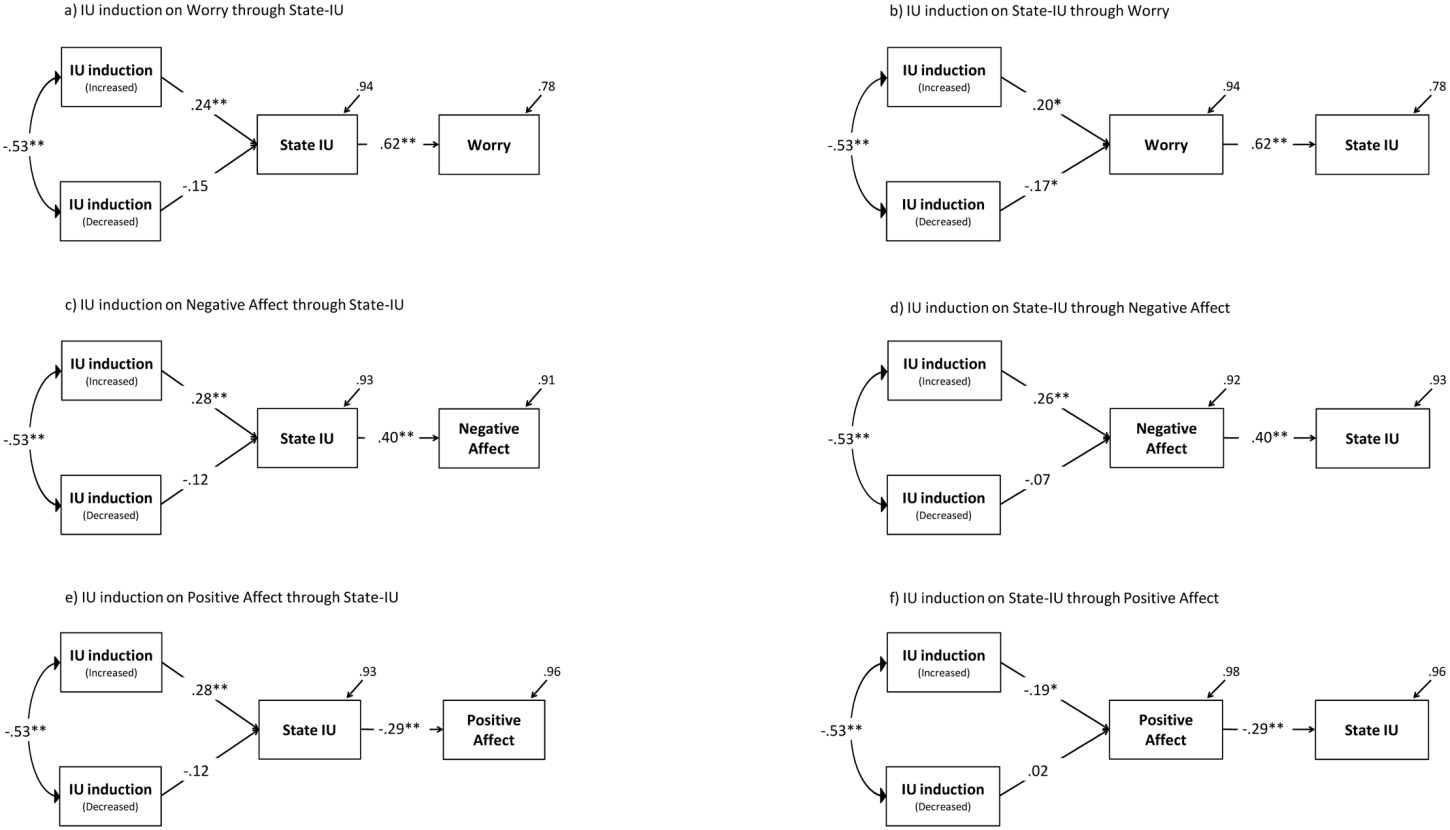
Mediation analysis.

In keeping with the IUM [2,37], mediation analyses were first conducted with state-IU and Worry as endogenous variables (Fig 4). The associated fit indices supported the model with state-IU as a mediator (χ^2^ = 4.12; *df* = 2; *p* = .13; CAIC = -8.10) more so than an alternative model with Worry as the mediator (χ^2^ = 6.27; *df* = 2; *p* < .05; CAIC = -5.95). Likewise, the fit indices supported a model assuming state-IU as mediator (χ^2^ = 6.16; *df* = 2; *p* = .04; CAIC = -6.06) more so than the alternative model with Negative Affect as the mediator (χ^2^ = 12.37; *df* = 2; *p* < .001; CAIC = 0.14). Finally, the fit indices also supported a model with state-IU as mediator (χ^2^ = 1.98; *df* = 2; *p* = .37; CAIC = -10.16) more so than the alternative model with Positive Affect as the mediator (χ^2^ = 18.51; *df* = 2; *p* < .001; CAIC = 6.28). Models with State-IU as mediators had a better fit than models reversing the order of mediator and dependent variables, thus expanding the IUM beyond the clinical settings to the community.

## Discussion

IU induction instructions for the current experiments were adapted from French to Italian following Grenier and Ladouceur [25], who carried out a similar investigation with Canadian participants. Specifically, Grenier and Ladouceur [25] manipulated IU in a two-stage experiment and used IU induction statements, read by the experimenter and then repeated aloud by the participant, with a neutral fictitious event (i.e., imagining to have ingested a medication). In the current experiments IU was induced by having participants consider (Study 1) or review (Study 2) an idiosyncratically-selected potential negative event–instead of a fictitious neutral event–and having participants self-administering the manipulation on the same day (Study 1) or 7-to-14 days after pre-test (Study 2), in which they had disclosed the negative event.

Temporary changes on IU-states were consistently reproduced across studies. Specifically, people receiving instructions designed to increase IU reported greater IU, Worry, and Negative Affect than those receiving instructions designed to decrease IU, and than those in a control condition, receiving merely diversionary ones; nevertheless, participants receiving instructions designed to decrease IU were not statistically different from those in the control condition. These findings replicated Grenier and Ladouceur [25], wherein increased IU was only reported by participants with low scores on situational anxiety at pre-test, a condition that was also likely to occur for our student sample participating to experiment as part of their course. Being able to increase but not decrease IU, Worry, and Negative Affect under tightly controlled experimental conditions contrasts previous clinical research [17,19,18]; however, the contrast may result from floor effects, likely to occur in a non-clinical sample with relatively low IU scores.

The current results extend IU theory [5,37] in several ways. First, reading statements designed to decrease IU or to divert attention from uncertainty; as such, non-clinical participants may have spontaneously decreased Worry and Negative Affect elicited by the personal negative event. Second, research participants, otherwise tending spontaneously to decrease Worry and Negative Affect, temporary acquired a IU mindset reading-through specific core beliefs statements (e.g., uncertainty is unacceptable and should be avoided; uncertainty is stressful and upsetting’ or being uncertain is unfair) [41], which in turn increased event-related Worry and Negative Affect. Accordingly, the current conclusions are consistent with the view that IU beliefs influence worry in non-clinical and clinical populations [5,37].

Mediation analyses carried out by exploratory (Study 1) and confirmatory (Study 2) approaches, showed statistically significant indirect effects of IU induction instructions on changes in Worry and Negative Affect, which both appear mediated by changes in state-IU. In particular, all models that represented the mediation chain from IU induction to state Worry, Positive Affect, or Negative Affect through state IU always had a better fit to the data (and likely approximating a true model) than models reversing dependent variables and the mediator. This findings parallel clinical research, showing that changes in IU often preceded changes in Worry over the course of treatment and not the other way around [41, 42, 43, 17, 44]. The results are also consistent with recent trends in personality literature wherein affective traits, like IU, are related to consistent pattern of behavior, cognition, and goal pursuit across time and situations [45, 46].

The methodological contribution expanded on Grenier and Ladouceur [25] in two ways. First, experimenters did not require cognitive-behavioral training to induce specific IU states. Second, participants did not require extensive interviewing. Accordingly, pending independent replication, the methods can now be used in broader, non-clinical contexts. Indeed, the standardized procedure can be used to produce temporary IU mindsets related to personal negative events as well as to potential negative outcomes during specific tasks (e.g., failing to provide a correct answer based on incomplete or missing information); accordingly, the changes may produce detectable effects on task performance or on attitudes towards the task itself (e.g., poor problem orientation; [47]), that can be later assessed by comparing research participants on meaningful outcomes after being exposed to an increasing IU condition verses a control condition (e.g., waitlist; neutral).

The interplay of IU, anxiety, and cognitive performance has traditionally been studied by relating self-reported IU scores to cognitive processing and affective states during experimental tasks (e.g., recalling uncertainty-related words, [48]; overestimating arousal on probability judgment tasks, [49]). An implication of our finding is that increasing IU may be relevant to affect performance on problem solving and decision-making tasks involving discount of future consequences (e.g., inter-temporal choices), risk perception (e.g., increased likelihood of negative outcomes and overestimation of their perceived costs, [50]) or risk-taking [51–53]. For instance, two recent studies have shown that high trait-IU is related to disadvantageous choices when an uncertain greater reward is delayed in time relative to a smaller immediate reward [10,21]. On the other hand, however, other recent studies have shown that both IU and worry can be effective ways to cope with uncertain aversive events in laboratory settings [54,55]. These studies were all based on self-report trait-IU measures, and in some cases involved comparisons with clinical samples. Instead, researchers interested in judgment and decision making or experimental social psychology would benefit from being able to experimentally increase IU, irrespective of clinical status. Next studies should then test whether increasing IU instructions, which were effective to increase worry for a personal negative event, can also increase worry for future outcomes in behavioral decision tasks or in real life health decisions.

Key limitations in the current studies also provide directions for future research. First, despite the generally consistent results across the two studies, both studies used self-report data collected from undergraduate psychology students. Student data may involve a selection bias associated with personality characteristics and demographic variables that may relate to IU, such as lower extraversion and conscientiousness, or younger age and predominantly female gender [56]. These specific characteristics might have introduced uncontrolled systematic variance components in our experiments; accordingly, the results may not generalize to other populations. Second, the experimental manipulation and the outcome measures both belonged to the same semantic domain, uncertainty, potentially limiting generalizability. Accordingly, a word priming effect may have occurred for state-IU questionnaire items. Future research should attempt to replicate the current results using behavioral assessments, implicit measures, or physiological measures, such as an Implicit Association Test [57], an Affect Misattribution Procedure [58], or monitoring participants’ heart rate variability. Indeed, previous research has suggested a critical relationship between uncertainty and heart rate variability [59]. Last, although the VAT procedure was used in pre-test to collect participants personal negative events and to assess IU-related beliefs and affects, we did not check whether this procedure affected participants baseline IU, Worry or Negative Affect ratings. Some participants may have been greatly affected by the VAT, and others may not have been affected at all. These individual differences might have biased our pre-test data, introducing potentially systematic error variance. Even if we controlled for pre-test scores in data analysis, future research should address this issue through additional manipulation checks. Last, it is worth mentioning that we did not assess research participants at any further time point. So we cannot say how long did the increased states of IU last. For those interested in inducing a temporary IU mindset on subsequent probability judgments or behavioral decisions, a very short-term IU increase can be enough. Again, future research might help disentangling this issue setting an additional follow up.

Notwithstanding the aforementioned limitations, the current studies validated an IU manipulation procedure for reliably inducing changes in state-IU within cognitive processing investigations. The results supported the efficacy of the procedure for experimental research. Subsequent research on IU may now use the procedure to assess for causal relationships between IU and perceptual and social judgments. We expect the results of such research will further demonstrate the importance of IU as a multidisciplinary construct affecting clinical and non-clinical cognitive processes.

## Supporting Information

S1 Appendix Self-administered Vertical Arrow Technique. (PDF)

S2 Appendix Statements used for (a) increasing or (b) decreasing participants intolerance of uncertainty or (c) control condition. (PDF)

S3 Appendix Statistical analyses.1. (PDF)
