# Supplementary material for: Intolerance of Uncertainty: A Temporary Experimental Induction Procedure
Source: PLoS One. 2016 Jun 2;11(6):e0155130. doi: 10.1371/journal.pone.0155130 (PMC4890765; doi:10.1371/journal.pone.0155130)
Supplement: S1 Appendix — (PDF) [file pone.0155130.s001.pdf]

S1 Appendix. Self-administered Vertical Arrow Technique

Imagine a potential negative future event that make you feel anxious:

If the negative event that you imagine would take place, what it could happen? (Write down *until a maximum of three possible consequences in the boxes below*)

Consequence 1:

If the consequence 1 would take place, what it could happen? (Write down until a maximun of three possible consequences in the boxes below)

Consequence 2:

If the consequence 2 would take place, what it could happen? (Write down until a maximun of three possible consequences in the boxes below)

Consequence 3:

If the consequence 3 would take place, what it could happen? (Write down until a maximun of three possible consequences in the boxes below)

Consequence 1a:

Consequence 1b:

Consequence 1c:

Consequence 2a:

Consequence 2b:

Consequence 2c:

Consequence 3a:

Consequence 3b:

Consequence 3c:
