# Supplementary material for: Intolerance of Uncertainty: A Temporary Experimental Induction Procedure
Source: PLoS One. 2016 Jun 2;11(6):e0155130. doi: 10.1371/journal.pone.0155130 (PMC4890765; doi:10.1371/journal.pone.0155130)
Supplement: S2 Appendix — (PDF) [file pone.0155130.s002.pdf]

S2 Appendix. Statements used for (a) increasing or (b) decreasing participants intolerance of uncertainty or (c) control condition.

a) Increased IU condition.

With respect to the negative event pointed out, it's difficult to not know what will happen; It's frustrating to not know what will happen to me; I don't know what will happen to me; It's out of my control; This is exactly what bothers me, I love to have everything in control; I don't know what will happen; It's bothersome not to know it; It's difficult to live with fullness of possibilities; Actually, with respect to the negative event pointed out there is more than one possibility; I don't know what will happen to me; It's difficult to live in this uncertainty climate; I don't feel good in everything that is uncertain; It's unacceptable to live like this; I don't know what will happen; I should be able to organize and plan everything beforehand, but I don't know what will happen in the future; It's difficult to not know; Unexpected events bother me enormously; It is not fair to have no guarantees in life; I don't know what is going to happen to me; I am not able to live in this uncertainty climate; It's difficult to not know what will happen to me; It's difficult to live with a lot of possibilities; It doesn't matter what I do, future is always full of uncertainties and I lose control; With respect to the negative event pointed out, I don't know what will happen to me; It's frustrating to not know it, it's disturbing to not know it; Zounds! I don't know what will happen to me; It's difficult to live in this uncertainty climate; I don't feel at ease in everything that is uncertain; It's difficult to live with different possibilities; Indeed, with respect to the negative event pointed out, there are different possibilities and unfortunately I don't know what will happen to me; It's difficult to live in this climate; I don't feel good in everything that is uncertain; It's inadmissible to live like this;

b) Decreased IU condition.

With respect to the negative event pointed out, I have to live with different possibilities; This is part of life; It doesn't disturb me to not know what will happen to me; I will see in due time; I have to live day by day, one day at a time; It is the same for all, the only things that are certain are those that have occurred in the past; It is like this and I cannot do anything about it; It doesn't disturb me to not know what will happen to me; I have to live day by day, one day at a time; Whatever happens, I leave the things flow, as I will see in due time what will happen to me; No one is able to plan and organize everything beforehand; I am able to face life even if everything is uncertain; Unexpected events don't bother me; I have to live with different possibilities; This is part of life; This is the same for everyone; Everyone has to live with this kind of possibilities; With respect to the negative event pointed out it's not important what I make, future will always have a degree of uncertainty; Anyway, this uncertainty doesn't scare me; Whatever happens, let the things flow, it is the best thing to do; It's impossible to know what will happen in the future; I have to live day by day, one day at a time; I will see what will happen to me; It doesn't bother me to not know what will happen to me; I will see in due time.

c) <u>Control condition.</u>

Tokyo is the most populous metropolitan area in the world. Inhabitants are 34.900.000; The second most populous metropolitan area in the world is New York. Inhabitants are 21.600.000; The third most populous metropolitan area in the world is Seoul. Inhabitants are 21.150.000; The least populous state in the world is the Vatican City with its 541 inhabitants; The second least populous state in the world is Tuvalu with its 9981 inhabitants; Only 5% of births take place on the scheduled day; Every human being has, besides the fingerprints, also imprints on the surface of the tongue that are unique and identifiable; Recent studies have shown that to wash the hands properly you need at least 20 seconds; Every single drop of blood covers its path in the human body in approximately 20 seconds; Nose and ears of every human being continue to grow throughout their life; The longest river in the world is the Amazon River with 6,937 km of length; The second longest river in the world is the Nile with 6,695 km of length; The coldest place in the world is the Vostok station in Russia (-89.2 ° C); The warmest place in the world is located in Death Valley in California (56.7 ° C); The tallest building in the world is the Burj Khalifa in Dubai (838 meters); The oldest museum in the world is the Royal Armouries Museum which is located inside the Tower of London; it was opened to the public in 1660; The wettest place on earth is Masynram, in Meghalaya, India. Each year there are 11,873 millimeters of precipitation; The most expensive journey in the world was a visit to the International Space Station and it cost 26 million euro; The fastest man in the world on the long distance is generally considered the world marathon record holder (42 kilometers and 195 meters); The current holder is the Kenyan Dennis Kipruto Kimetto; The highest speed attained by a man by jumping into the atmosphere from an altitude of 38 969 m is of 1,357 km / h; The higher speeds officially registered on the short distance are calculated starting from still on 100 and 200 meter dash; The current record holder is the Jamaican Usain Bolt; The oldest woman in the world lived for 122 years and 164 days; The oldest man in the world lived for 116 years and 54 days; The tallest man in the world measured 2.72 meters and weighed 220 kg.
