# Supplementary material for: Intolerance of Uncertainty: A Temporary Experimental Induction Procedure
Source: PLoS One. 2016 Jun 2;11(6):e0155130. doi: 10.1371/journal.pone.0155130 (PMC4890765; doi:10.1371/journal.pone.0155130)
Supplement: S3 Appendix — (PDF) [file pone.0155130.s003.pdf]

S3 Appendix.

STATISTICAL ANALYSES

STUDY 1.

Analysis of Covariance. A series of ANCOVAs were conducted on post-test QIII subscales scores as well as on post-test PANAS Positive and Negative Affect scores by experimental condition, controlling for the respective pre-test scores. This strategy is strongly recommended for analysis of pre-post test data to reduce within-group error variance and to remove potential confounding factors, such as differences on pre-test scores or carryover effects in within-subject designs [1]. Given the relatively low sample size, we assessed parametric assumptions for ANCOVA, including normal distribution and constant variance of dependent variables and covariates. Preliminarily, however, all variables were pre-screened for potential outliers through visual inspection of Box-Whisker diagrams. An outlier is a case whose value is smaller than the 25th percentile minus 1.5 times the inter-quartile range, or larger than the 75th percentile plus 1.5 times the inter-quartile range. As such, the Box-Whisker plot does not use potential outliers in the computation of spread, which is based on percentiles rather than on variance. Three cases were identified as outliers and removed from subsequent analyses. As seen in Table 1, the Shapiro-Wilks statistic - a normality test for small sample data - was significant for Negative Affect scores, both at pretest and post-test and for IU at post test. Skeweness and kurtosis values were around the normal range (i.e., ±1.00), thus showing minor deviations from normality. The Levene test for homogeneity of variances between experimental conditions was never significant. Given these minor, but significant, violations of parametric assumptions, research hypotheses were re-examined based on a distribution-free method for analysis of covariance. “The recommended procedure in experimental designs for which no nonparametric test exists is to use the usual analysis of variance on the data and then to use the same procedure on the rank transformed data” [2]. In our specific case, we first

ranked both post-test and pre-test score using SPSS Rank procedure. Second, each rank-transformed dependent variable was regressed on the rank transformed covariate, saving un-standardized residuals. Last, a one-way ANOVA of residuals by experimental condition was carried out. This non-parametric analysis, referred to as Quade's rank transformation method or RANCOVA, is deemed robust from deviations from parametric assumptions and preserves the nominal significance level [3,4].

Mediation analysis. Mediation analyses with manifest variables were carried out by INDIRECT SPSS procedure. Specifically, we assessed the indirect effect of IU induction on state-Worry and state-affect through state-IU as the product of coefficients linking the independent variable (i.e., IU induction) to mediator (e.g., state-IU) and mediator to each dependent variable (e.g., state-Worry), respectively (Fig 2a). Importantly, pre-test scores for mediator and dependent variables were used as covariates. As the product of coefficients method had non-normal sampling distribution - nor the collected data fully met all parametric assumptions - the significance test for each indirect effect displayed in Fig 2 was based on bootstrap bias corrected confidence intervals. Confidence interval (CI) for each product of coefficients that does not include zero support the statistical significance of the hypothesized mediation. Each analysis used 5000 bootstrap replications, each producing an indirect effect of independent variable on the dependent variable through mediator. Importantly, the bootstrap approach is inherently non parametric, as it re-samples with replacement from the collected data and not from the normal distribution [5].

Study 2.

Analysis of Covariance. Like Study 1, ANCOVAs were conducted on post-test QIII subscales scores as well as on post-test Mood Adjectives affect scores controlling for the respective pre-test scores as well as for the time interval between Session 1 and Session 2. Despite larger sample size than Study 1, we also tested the parametric assumptions for ANCOVA after cleaning up the data for potential outliers. As in Study 1, two cases were identified as outliers based on inspection of Box-Whisker plots and removed from subsequent analyses. As seen in Table 1, the

Shapiro-Wilks statistic was significant for Negative Affect scores, both at pretest and post-test and for Positive Affect and Worry at post-test. However, skeweness and kurtosis values were in the normal range for all variables. Likewise, the Levene test for homogeneity of variances between experimental conditions was never significant. For the sake of prudence, however, research hypotheses were re-examined based on RANCOVA.

Mediation analysis. Unlike Study 1, mediation relations were tested through confirmatory path analysis models with manifest variables carried out by EQS 6.1 [6]. Using this method, different regression relationships were simultaneously modeled and each model was assessed in terms of discrepancy between observed data and those expected assuming the hypothetical model (i.e., goodness of fit). Experimental conditions were modeled as exogenous dummy variables (i.e., Dummy 1 = increasing IU vs. decreasing IU and control; Dummy 2 = decreasing IU vs. increasing IU and control) [7]. To examine whether the changes in worry and affect measures were attributable to the aforementioned exogenous variables trough changes in state-IU, residual change scores were computed for each endogenous variable in the model, controlling for each other. In particular, the residual change score is the component of the post-test score that could not be predicted from pre-test scores. Different from raw change scores, residual change scores are independent of pre-test scores and preserve high reliability even if the correlations between pretest and post test are high [8]. Multivariate normality assumptions for Maximum Likelihood estimation were preliminarily tested through inspection of the Mardia's normalized coefficient. Values greater than 3.00 are deemed indicative of non-normality [9]. In our specific case the assumptions were substantially met; Mardia's coefficients were 2.67, 3.02 and 2.65 respectively for models a) and b), c) and d), e) and f), respectively (see Fig 4). As in Study 1, testing for mediation using data with mediator and dependent variables assessed at the same point in time might creates a confound for interpretation. We specified alternate mediation models for each dependent variable. Types of models were non-nested (i.e., not all parameters of one model are included in the other model), nonequivalent (i.e., models cannot fit the data equally well), and differed as follows: 1) Type 1 models (i.e., panels a, c

and e, in Fig 4) represented the mediation chain from IU induction to state-Worry, Positive Affect, and Negative Affect through state-IU; 2) Type 2 models (i.e., panels b, d and f, in Figure 4) reversed the mediation change so that state-IU became the dependent variable, while each of the other endogenous variables were set as mediators (see Fig 4). Alternative models were compared to identify the one with maximum generalizability; as such, Consistent Akaike Information Criterion (CAIC) was used as the comparator index. Smaller CAICs are associated with higher likelihoods that the tested model approximates the true model. CAIC is easy to calculate and more robust than other same class indexes, such as Akaike Information Criterion or Bayesian Information Criterion [10].

References

1. van Breukelen, GJ. ANCOVA versus CHANGE from baseline in nonrandomized studies: The difference. Multivariate Behav Res, 2013; 48(6):895-922.

2. Conover WJ. Practical nonparametric statics. John Wiley & Sons, Inc., New York, 1999; 130-133.

3. Bonate PL. Analysis of pre-test-posttest designs. CRC Press; 2000.

4. Wu XW, Lai D. Comparison of Statistical Methods for Pre-test–Posttest Designs in Terms of Type I Error Probability and Statistical Power. Commun Stat Simul Comput, 2015; 44(2): 284-294.

5. Preacher KJ, Hayes AF. Asymptotic and resampling strategies for assessing and comparing indirect effects in multiple mediator models. Behav Res Methods, 2008; 40(3): 879-891.

6. Bentler PM. EQS 6 structural equations program manual. Encino, CA: Multivariate Software; 2004.

7. Tabachnick BG, Fidell LS. Multivariate analysis of variance and covariance. Using multivariate statistics 3, 2007; pp. 402-407.

8. Allison PD. Change scores as dependent variables in regression analysis. Sociol Methodol, 1990; 20(1): 93-114.

9. Ullman JB. Structural equation modeling: Reviewing the basics and moving forward. J Pers Assess, 2006; 87(1): 35-50.

10. Hoyle RH. Handbook of structural equation modeling. Guilford Press; 2012.
